# Rapidly Growing and Aggressive Squamous Cell Carcinoma of the Forearm: A Report of Successful Treatment With Mohs Surgery and Complex Reconstruction With Rhombic Triple Z-Plasty

**DOI:** 10.7759/cureus.55182

**Published:** 2024-02-28

**Authors:** Harib H Ezaldein

**Affiliations:** 1 Mohs Micrographic Surgery, Bennett Surgery Center, Santa Monica, USA; 2 Dermatology, Miami Dermatology and Mohs Surgery, Miami, USA

**Keywords:** dermatopathology, nonmelanoma skin cancer, hepatic tumors, clinical dermatology, immediate reconstruction, plastic surgery, squamous cell carcinoma of the skin, eruptive keratoacanthoma, mohs surgery

## Abstract

Reconstruction of complex post-surgical wounds requires functional and aesthetic considerations. We present a case of a complex radial-dorsal forearm defect in a patient who underwent Mohs surgery for an aggressive and rapidly growing squamous cell carcinoma. Following complete tumor excision, we utilized a modified rhombic flap for complete wound coverage with long-term conservation of extensor function. The rhombic flap modification included three Z-plasties at the flap base to add rotational components to the flap transposition. Long-term follow-up showed acceptable cosmesis, preserved extensor tendon function, and no evidence of tumor recurrence.

## Introduction

Mohs micrographic surgery (MMS) is a specialized skin cancer treatment with a high cure rate due to complete peripheral and deep marginal assessment [1]. Although MMS is beneficial in treating high-risk tumors that extend beyond standard excisional margins, the resulting surgical defects can be large and expose deeper vital structures, and thus be challenging to reconstruct [2,3]. Many considerations must be accounted for in a surgeon’s approach to complex reconstruction such as the exposing of vital underlying structures, free margin distortion, and cosmetic unit delineations, among others. This report highlights the repair of a deep and extensive surgical defect surgery with exposed vital structures with an adjacent skin flap, under local anesthesia.

## Case presentation

**Figure 1:**
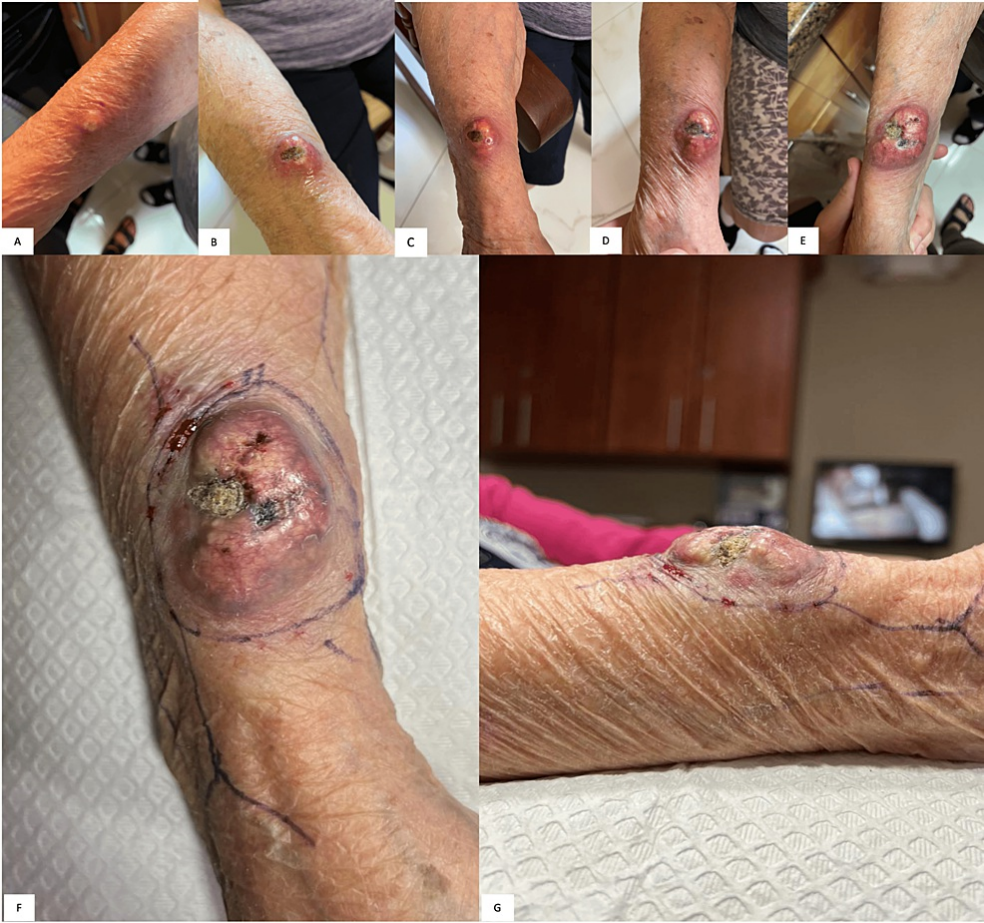
Preoperative appearance of our patient’s tumor Her lesion progressed from a pink to red papule with erosion secondary to traumatic scratching, to an ulcerated, keratotic nodule with symptoms of throbbing pain and impetiginization over four months (A-E). She presented with a 5 cm, seemingly non-mobile tumor at the time of surgery with exophytic growth consistent with a high-grade clinical keratoacanthoma (F-G). The initial margin of 1 centimeter and visible bridging veins were marked prior to surgical removal.

An 81-year-old female presented for surgical removal of a squamous cell carcinoma that rapidly grew to a size of 5.1 cm x 3.5 cm over four months. Initially presenting as a dark pink to red papule measuring only a few millimeters, the papule grew rapidly after the patient initially scratched it off, eventually developing into a painfully throbbing and ulcerated tumor on her right radial-dorsal forearm (Figure 1). She was seen by her primary dermatologist who performed a thin shave biopsy and immediately referred her to our practice for emergent management. At the time of surgery, the patient did not show evidence of clinical lymphadenopathy or satellite, or in-transit, metastatic lesions.

**Figure 2:**
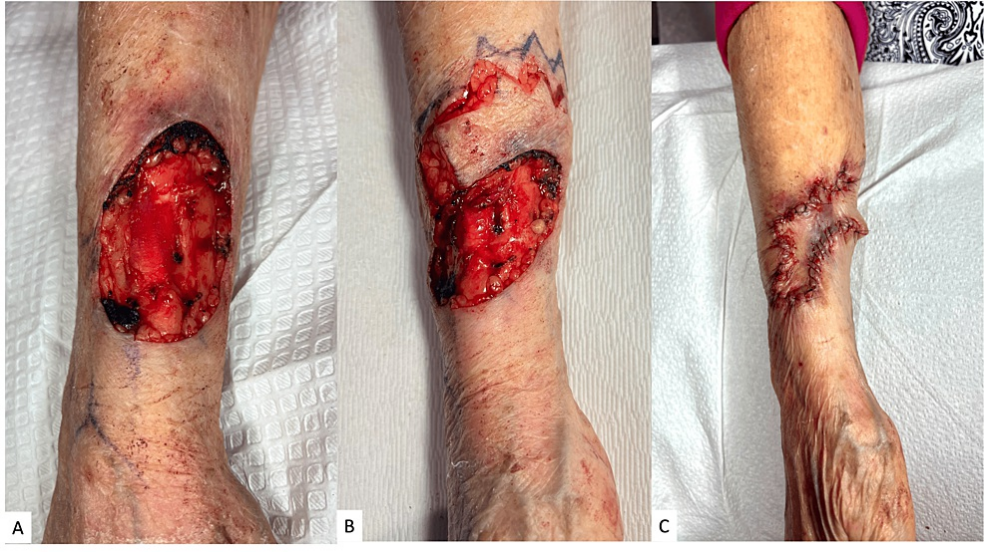
Surgical defect after tumor removal and immediate reconstruction Postoperative appearance of the surgical site with exposed periosteal tissue and exposed extensor tendons (A). Anterior view of the postoperative defect and incised rhombic flap with triple Z-plasty (B). Immediate post-reconstruction photograph with the flap carefully sewn into the defect (C).

**Figure 3:**
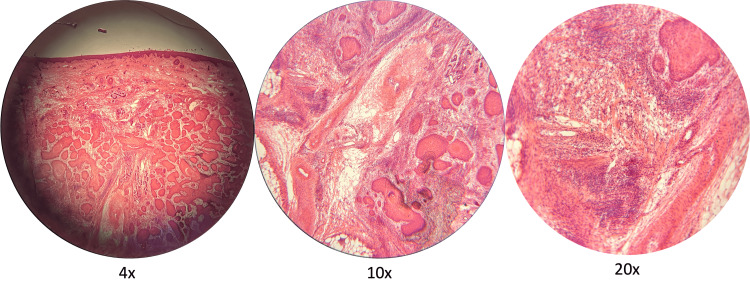
Appearance of the patient's squamous cell carcinoma tumor under a microscope There are moderate to well-differentiated tumor islands in the deeper levels of the dermis and around the base of adnexal structures at 4x magnification. At 10x, the islands of squamous cells are seen beyond the superficial adipose layers with proximity to neurovascular bundles. At 20x, the dense inflammation around small caliber cutaneous nerves is seen without direct evidence of perineural invasion.

The patient’s tumor was removed with MMS after three surgical removal attempts, with a final surgical defect, measuring 7.0 cm x 5.4 cm, that extended deep to the periosteum and had exposed extensor tendons, both of which were free of tumor involvement (Figure 2). The patient notably demonstrated strong extensor functions after tumor removal and prior to reconstruction. Histological findings were significant for the infiltration of the tumor into deeper layers of the dermis and beyond the subcutaneous fat, accompanied by dense lymphohistiocytic inflammation at and around well-differentiated islands of squamous cell carcinoma (Figure 3). These findings are consistent with a high-grade keratoacanthoma with well-differentiated invasive squamous cell carcinoma, staged as a T2B per Brigham staging system, widely recognized as the standard criteria among surgeons for evaluating these types of tumors [4].

The approach to these defects must rely on factors such as a patient’s skin laxity and tension placed on the closure secondary to nearby dynamic free margins such as this patient’s wrist or finger movements. Skin grafts were a consideration, however, the high failure rate for large full-thickness skin grafts and the poor cosmetic appearance of split-thickness ones precluded their use [5]. Healing with secondary intent or with a partial purse string was also considered; however, the high risk of postoperative bleeding in this patient and possible tendon damage or rupture were important risk factors [6].

After careful consideration of various treatment options, a rhombic flap with a triple Z-plasty at its base was designed for coverage of the defect. This flap was designed at a 90-degree takeoff angle with rhombic limbs at 60 degrees, followed by 135 degrees, 105 degrees, and 90 degrees for the first, second, and third Z-plasties, respectively (Figure 3).

Additionally, three Z-plasties were serially incised proximal to the rhombic flap. Adequate undermining was performed on all edges of the primary and secondary defects. The flap was then rotated and transposed into the defect and secured by subcutaneous 4-0 polyglactin-910 sutures and epidermal 5-0 polypropylene sutures in a simple interrupted and simple continuous fashion (Figure 3c). The patient’s extension function was maintained postoperatively, and a pressure dressing was placed. An arm board was unavailable in the clinic but has been shown to aid with the survival of similar flaps [5].

**Figure 4:**
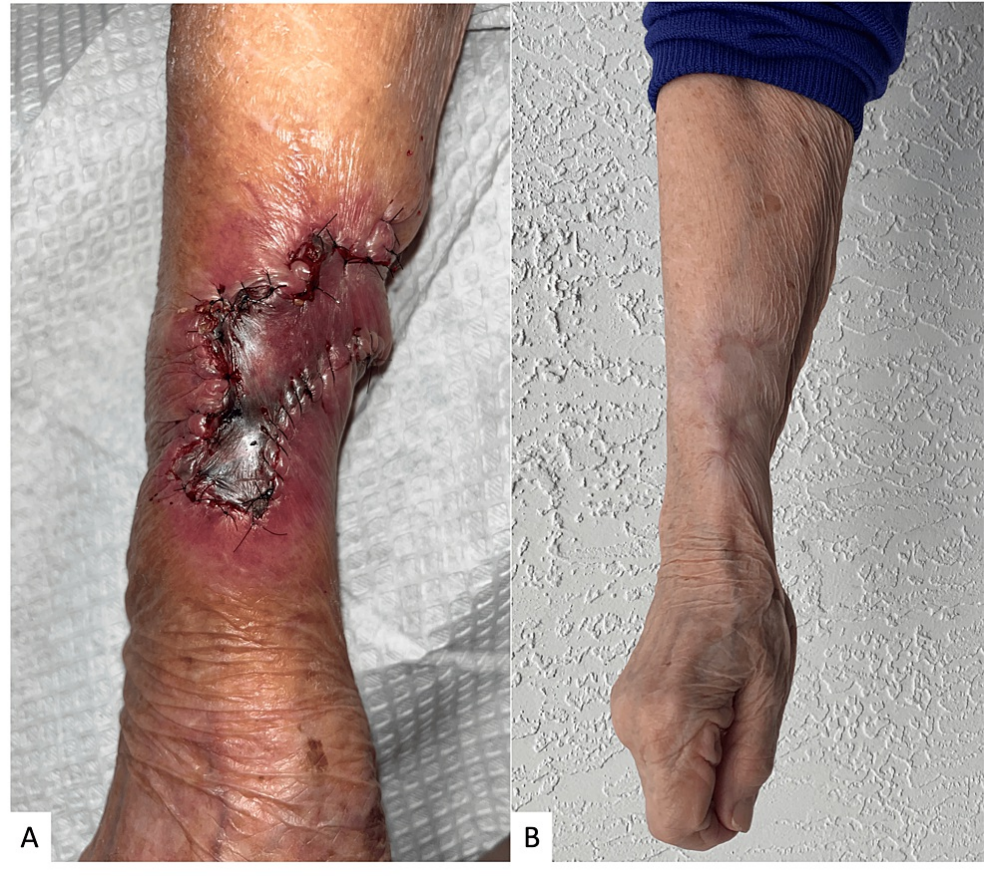
Post-reconstructive follow-up and final result Follow-up at one week was significant for a venous congestive and ecchymotic appearance (A). Her six-month result postoperative result was significant for slight erythema at incisions but otherwise for full extension strength and no evidence of recurrence.

When seen at the following follow-up appointments, some initial venous congestion was noted on postoperative day 7, which improved and resulted in an acceptable final appearance (Figure 4). There was no evidence of tumor recurrence at nine months postoperative follow-up.

## Discussion

In the reconstructive approach to forearm defects, one must consider vascular anatomy, skin tension during pronation and supination, coverage for underlying vital structures, and the presence of dynamic free margins such as mobile joints. Our case featured the presence of vital structures, however, most were protected by fascial linings, thereby precluding the need for any protective repair. Thus, our repair was primarily for skin and subcutaneous coverage.

Various coverage options exist such as skin grafts, adjacent advancement or rotation flaps, or vascularized free flaps. Local random pattern flaps have the advantage of predictability and cost-effectiveness. Rotation flaps would need to originate from the posterior aspect and can create significant secondary tension, while advancement flaps would not be an effective option from the lateral or medial aspects of the defect. A transposition approach allowed us to keep the secondary tension on the volar surface of the forearm and maintain a vascular bed. Furthermore, the use of the additional Z-plasties allows for additional advancement or rotational components for flap movement [7].

Although granulation is an option, the risk of tendon rupture or deep structure injury was considered. The disadvantage to flap or graft reconstruction lies in the difficulty of determining tumor recurrence for deep-seated tumors. Although the Mohs technique uses a full-margin assessment of the tumor margins, it is entirely possible that certain skin tumors may have clinically undetectable satellite tumors or already have a subclinical lymphovascular presence [8]. Despite the lack of perineural invasion on histology and postoperative positron emission tomography (PET)-computed tomography (CT) scans negative for locoregional or metastatic disease, our practice still recommends adjuvant radiation and close monitoring for high-grade skin cancers, even when reconstructed. Often, a deep-seated tumor may also be difficult to asses clinically, illustrating an advantage to postoperative radiation treatment [9]. 

Our patient was placed on a four to six-month follow-up schedule for the first two years, eventually extending to twice a year for the next three, and then back to yearly skin exams. Although the U.S. Preventive Services Task Force (USPTF) no longer supports the utility of skin cancer screenings in the general population, we still recommend such screening for the earlier detection of skin tumors, as supported by several studies in the dermatologic literature [10-11].

## Conclusions

Deep forearm defects can be challenging to reconstruct. A surgeon must consider vascular supply, coverage of underlying critical structures, and the presence of dynamic free margins when choosing a reconstructive approach. Here, we demonstrate a successful use of a local tissue flap for covering a large surgical defect following a successful Mohs surgery for a high-grade squamous cell carcinoma.
